# Transcriptional activation of CBFβ by CDK11^p110^ is necessary to promote osteosarcoma cell proliferation

**DOI:** 10.1186/s12964-019-0440-5

**Published:** 2019-10-14

**Authors:** Yong Feng, Yunfei Liao, Jianming Zhang, Jacson Shen, Zengwu Shao, Francis Hornicek, Zhenfeng Duan

**Affiliations:** 1 Department of Orthopaedic Surgery, Wuhan Union Hospital, Tongji Medical College, Huazhong University of Science and Technology, 1277 Jie Fang Avenue, Wuhan, 430022 China; 2 Sarcoma Biology Laboratory, Department of Orthopaedic Surgery, Department of Orthopaedic Surgery, David Geffen School of Medicine at UCLA, 615 Charles E. Young Dr. S, Los Angeles, CA 90095 USA

**Keywords:** CDK11, CBFβ, Osteosarcoma, Transcription

## Abstract

**Background:**

Aberrant expression of cyclin-dependent protein kinases (CDK) is a hallmark of cancer. CDK11 plays a crucial role in cancer cell growth and proliferation. However, the molecular mechanisms of CDK11 and CDK11 transcriptionally regulated genes are largely unknown.

**Methods:**

In this study, we performed a global transcriptional analysis using gene array technology to investigate the transcriptional role of CDK11 in osteosarcoma. The promoter luciferase assay, chromatin immunoprecipitation assay, and Gel Shift assay were used to identify direct transcriptional targets of CDK11. Clinical relevance and function of core-binding factor subunit beta (CBFβ) were further accessed in osteosarcoma.

**Results:**

We identified a transcriptional role of protein-DNA interaction for CDK11^p110^, but not CDK11^p58^, in the regulation of CBFβ expression in osteosarcoma cells. The CBFβ promoter luciferase assay, chromatin immunoprecipitation assay, and Gel Shift assay confirmed that CBFβ is a direct transcriptional target of CDK11. High expression of CBFβ is associated with poor outcome in osteosarcoma patients. Expression of CBFβ contributes to the proliferation and metastatic behavior of osteosarcoma cells.

**Conclusions:**

These data establish CBFβ as a mediator of CDK11^p110^ dependent oncogenesis and suggest that targeting the CDK11- CBFβ pathway may be a promising therapeutic strategy for osteosarcoma treatment.

**Graphical Abstract:**

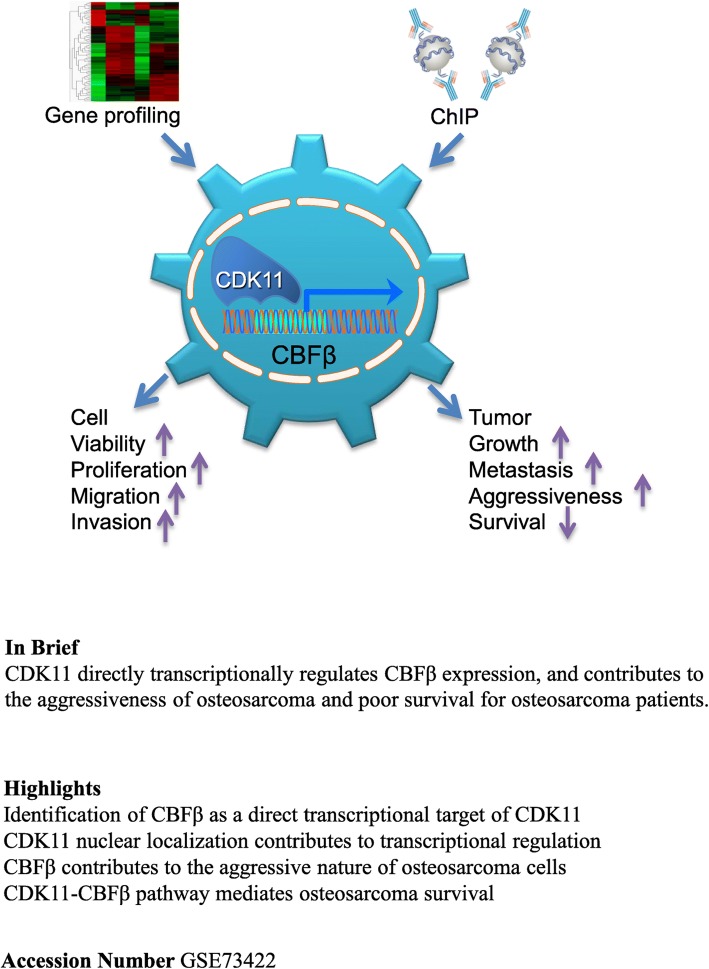

## Background

It has been well established that overexpression and activation of cyclin-dependent kinases (CDK) is a hallmark of human cancer [1, 2]. The Food and Drug Administration (FDA) has approved the CDK4/6 inhibitors Palbociclib, Ribociclib and Abemaciclib for treating metastatic breast cancer. These inhibitors have also demonstrated promising antitumor potentials both as a monotherapy and in combined therapy in numerous clinical trials [3–6]. CDK11 is a member of the serine/threonine protein kinase family. CDK11 plays a crucial role in cell proliferation and growth of cancers, such as breast cancer, ovarian cancer, multiple myeloma, and sarcoma [7–12]. Knockdown of CDK11 by shRNA or siRNA inhibits cancer cell growth and induces apoptosis. In vivo administration of CDK11 siRNA was shown to reduce tumor growth in cancer xenograft mouse models [13, 14]. Importantly, nuclear CDK11 expression levels correlate with clinical prognosis in cancer patients, including breast cancer, ovarian cancer, and sarcoma [8–10, 13–15]. Downregulation of CDK11 also causes significant loss of cell viability and clonal survival in breast cancer, colon cancer, cervical cancer, multiple myeloma, and acute myeloid leukemia [9, 16–19].

There are three major CDK11 protein isoforms encoded from an identical mRNA: CDK11^p110^, CDK11^p58^, and CDK11^p46^. The CDK11^p58^ isoform is generated by an internal ribosome entry site sequence (IRES) in the same mRNA encoding the CDK11^p110^ isoform. CDK11^p46^ is generated by caspase dependent cleavage of CDK11^p110^ and CDK11^p58^ [20, 21]. In general, these protein isoforms regulate RNA transcription and processing, mitosis, and apoptosis. CDK11^p110^ is expressed in all phases of the cell cycle, while CDK11^p58^ is expressed transiently only during the G2/M phase of mitosis. Inhibition of CDK11 specifically suppresses RNAP II-dependent transcription and can be rescued by addition of purified CDK11 [22]. However, the molecular mechanisms and signaling pathways of CDK11 regulated genes in cancer cells are largely unknown.

In the present study, we performed a global transcriptional analysis using gene array technology to investigate the transcriptional role of CDK11 in osteosarcoma. We identified CDK11^p110^, but not CDK11^p58^, in the transcriptional regulation of core-binding factor subunit beta (CBFβ) expression in osteosarcoma cells, which is important for bone cell development and formation of the skeleton. Knockdown of CDK11 caused a strong decrease in the levels of CBFβ. High CBFβ expression correlated with CDK11 expression and contributed to reduced overall survival in osteosarcoma patients. Our data suggest that a direct link exists between the CDK11^p110^- CBFβ pathway and osteosarcoma cell growth and migration.

## Methods

### Cell lines

The human osteosarcoma cell lines U-2OS, MG63, SaOS, MNNG/HOS, and 143B were purchased from the American Type Culture Collection (Rockville, MD). The human osteosarcoma KHOS cell line was kindly provided by Dr. Efstathios Gonos (Institute of Biological Research & Biotechnology, Athens, Greece). The human osteoblast cells NHOst and HOBc were purchased from LonzaWallkersville (Walkersville, MD) and PromoCell GmbH (Heidelberg, Germany), respectively. The osteosarcoma cell lines were incubated in RPMI 1640 (Life Technologies, Grand Island, NY) supplemented with 10% fetal bovine serum. The osteoblast cell lines were cultured in osteoblast growth medium (PromoCell) with 10% fetal bovine serum.

### Synthetic CDK11 or CBFβ siRNA transfection

Validation of CDK11 or CBFβ knockdown phenotypes in osteosarcoma cell lines was conducted by transfection of synthetic human CDK11 (Applied Biosystems, Grand Island, NY) or CBFβ siRNA (Applied Biosystems). The siRNA sequence targeting CDK11 corresponded to coding regions (5′-AGAUCUACAUCGUGAUGAAtt-3′, antisense 5′-UUCAUCACGAUGUAGAUCUtg-3′) of the CDK11 gene. The siRNA sequence targeting CBFβ corresponded to coding regions (5′-CCGAGAGUAUGUCGACUUATT-3′, antisense 5′- UAAGUCGACAUACUCUCGGCT-3′) of the CBFβ gene. Transfection with siRNA was performed using Lipofectamine™ RNAiMAX (Invitrogen, Grand Island, NY) according to the manufacturer’s instructions. The non-specific siRNA oligonucleotide (Applied Biosystems) was used as a negative control. Medium was changed 24 h after transfection with RPMI 1640 supplemented with 10% fetal bovine serum. Total protein was extracted 72 h after CDK11 or CBFβ siRNA transfection.

### Global gene expression profiling and analysis

Total RNA was extracted from the cells using TRIzol reagent (Invitrogen, Carlsbad, CA) and submitted to Human Genome U133 plus 2 arrays for microarray. Microarray studies were performed on KHOS and U-2OS cells that were transfected with CDK11 siRNA or non-specific siRNA. To account for and eliminate biological noise, RNA was isolated from three distinct flasks of each cell line 48 h post siRNA transfection. Total RNA was extracted from the cells using TRIzol reagent (Invitrogen, Carlsbad, CA) according to manufacturer’s guidelines. The quality and size distribution of the RNA were assessed for microarray analysis. Each sample was separately hybridized on Human Genome U133 plus 2 arrays, according to the manufacturer’s guidelines (Affymetrix, Santa Clara, CA). Raw Affymetrix intensity measurements were background corrected, normalized, and summarized into gene expression measurements using dChip (dchip.org; https://www.hsph.harvard.edu/pqg/software/). The average expression value for each gene across the arrays was used to normalize the mRNA intensities. The CEL files were transformed into intensity information using the RMA normalization of GeneSifter (http://www.genesifter.net/web/qs_analysis.html) and Qlucore 3.0 (New York, NY), and fold change was used to select differentially expressed genes [23–25]. Differentially expressed gene identifiers were uploaded into a heat map. Data are available at the database of Gene Expression Omnibus (https://www.ncbi.nlm.nih.gov/geo/) with the access code GSE73422 [26, 27].

### Plasmid constructions

GFP-CDK11^p110^ and GFP-CDK11^p58^ plasmids were kindly provided by Dr. Régis Giet. GFP-CDK11^p110^ kinase-dead or kinase-active mutations were generated. The pEGFP-N3 plasmid was purchased from Clontech (Mountain View, CA) as plasmid transfection control.

### Luciferase assays

The human CBFβ promoter in pLightSwith_Prom plasmid (SwitchGear Genomics, Carlsbad, CA) encodes the wild type promoter sequence (1 kb upstream of the CBFΒ gene 5′-UTR). CDK11^p110^, CDK11^p58^, and CDK11^p110^ mutant plasmids were transfected with Lipofectamine™ 3000 (Invitrogen, Grand Island, NY). LightSwitch™ Promoter Reporter Vector (SwitchGear Genomics) was transfected as an internal transfection control. Cells were collected 48 h after transfection and lysates were prepared for luciferase assays following the manufacturers’ protocols (SwitchGear Genomics). Luciferase activity with LightSwitch Assay Reagents was measured by the Perkin Elmer Victor 3 Microplate Readers (Waltham, MA).

### Chromatin immunoprecipitation (ChIP) assays

ChIP assays were performed with the U-2OS and KHOS cell lines according to the manual from Cell Signaling Technology (MA). The CBFβ promoters were amplified using PCR. In brief, 2 × 107 cells were transfected with CDK11 siRNA. Forty-eight hours after transfection, cells were crosslinked in 1% formaldehyde for 10 min at room temperature. Crosslinking was terminated by incubation of cells with glycine solution for 5 min. Cells were washed and chromatin was sonicated to a length of 100–900 bp using a VIRSONIC 100 Ultrasonic Homogenizer (Woburn, MA), while samples were maintained on ice at 4 °C. Protein G Magnetic beads (SimpleChIP® Enzymatic Chromatin IP Kit (Magnetic Beads), Cell Signaling Technology, Danvers, MA) were washed with blocking solution and pre-coated overnight with anti-CDK11 antibody (sc-928, Santa Cruz Biotechnology, Santa Cruz, CA), anti-Histone H3 (SimpleChIP®Kit, Cell Signaling Technology) as a positive control, or IgG as a negative control. IP samples were incubated overnight at 4 °C with rotation. Beads were collected by magnetic attraction using a magnetic rack. After washing, protein-DNA complexes were eluted and crosslinks were reversed by incubating the suspensions for 2 h at 65 °C. DNA was purified using spin columns. ChIP DNA was analyzed with PCR using positive and negative controls. The ChIP signal was normalized to the input sample and total DNA content. ChIP PCR primers were designed in the proximal regions of CBFβ gene promoters, around genomic locations where potential CDK11-binding elements were located. Standard PCR method was performed following the manufacturer’s ChIP Protocol (Cell Signaling Technology). PCR products were confirmed by sequencing and compared with CBFβ promoters.

### Western blotting assay

Total protein lysates from the osteosarcoma cells were harvested by 1× RIPA Lysis Buffer (Upstate Biotechnology, Charlottesville, VA) plus complete protease inhibitor cocktail tablets (Roche Applied Science, IN, USA). Protein Assay Reagents (Bio-Rad, CA) and a SPECTRAmax Microplate Spectrophotometer from Molecular Devices (Sunnyvale, CA) were applied to evaluate the protein concentrations. The primary antibodies for CDK11 (1:1000 dilution) and β-actin (1:2000 dilution) were purchased from Santa Cruz and Sigma-Aldrich, respectively. Secondary antibodies IRDye® 800CW or IRDye® 680LT were purchased from LI-COR (Biosciences, NE). Western blot analysis was performed as previously reported (Duan et al., 2008). Membrane signals were detected using the Odyssey infrared imaging system. Quantification analysis of western blot values was performed with Odyssey software 3.0 (LI-COR Bioscience, NE).

### MTT cell proliferation assay

KHOS or U-2OS cell lines were transfected with CBFβ siRNA or non-specific siRNA. 3 × 103 cells were seeded into each well of 96-well plates. Complete growth medium without antibiotics was added into each well to a volume of 100 μl in triplicate. After 72 h of incubation, 20 μl of MTT (5 mg/mL, dissolved in PBS, Sigma-Aldrich) was added to each well and the cells were cultured for 4 h at 37 °C. The MTT formazan product was dissolved with acid isopropanol. The absorbance at a wavelength of 490 nm (A490) was measured on a SPECTRAmax Microplate Spectrophotometer from Molecular Devices (Sunnyvale, CA). All results were analyzed by GraphPad Prism 5 software (San Diego, CA).

### Wound healing migration assay

Migration activity was detected by multiple scratch wounds assay. In brief, 1 × 105 cells per well were plated into 12-well plates and transfected with CBFβ siRNA. Three parallel lines were made in confluent cell cultures with a 200 μl tip. After suspended cells were washed away with serum-free medium, the cultures were fed with complete medium again. The wounds were observed at 0, 8, and 24 h after wounding, and photographed via microscope (Nikon Instruments, Inc.). Three images were taken per well at each time point using a 10× objective, and the distance between the two edges of the scratch (wound width) was measured at 10 sites in each image. The cell migration distance was defined as the wound width at the 0 h time point minus the wound width at each time point and then divided by two.

### Matrigel invasion assay

The matrigel invasion assay was performed with the BD BioCoat™ Matrigel™ Invasion Chamber (Becton-Dickinson, MA) following the manufacturer’s instructions. CBFβ siRNA or non-specific siRNA transfected cells (5 × 104 cell/plate) were seeded into the upper chamber of each well in serum-free medium, and complete medium was put into the bottom chamber. The invasion chamber was incubated for 24 h at 37 °C under 5% CO2. Afterward, non-invading cells were removed by scrubbing from the upper surface of the membrane with cotton-tipped swabs. After washing the cells with medium, complete medium with 1 μg/mL Hoechst 33342 (Invitrogen) was used to stain nuclei of invading cells for 5 min. Images were acquired by a Nikon Eclipse Ti-U fluorescence microscope and phase contrast microscope equipped with a SPOT RT digital camera. The number of invading cells was counted in three images per membrane by microscopy using a 20× objective.

### Immunohistochemical staining on TMA

The expression levels of CBFβ and CDK11 were determined based on the immunohistochemistry protocol (Paraffin) from Cell Signaling Technology (Beverly, MA) as previously described (Zhang et al., 2013). Briefly, 5-μm-thick array sections were baked at 60 °C for 1 h, dewaxed with xylene (three times for 5 min each), transferred through 100% ethanol (twice for 5 min each), rehydrated through graded alcohol, and then immersed in deionized water for 10 min. Antigen retrieval was processed with Target Retrieval Solution (Dako, North America, Inc., CA). Afterward, the slide was washed with PBS twice for 5 min. Following the process of antigen retrieval, endogenous peroxidase activity was quenched by incubation in 3% hydrogen peroxide. Then, blocking solution (Cell Signaling Technology) was applied for 1 h at room temperature, and primary CBFβ or CDK11 antibody (1:50 dilution, in 1% bovine serum albumin PBS) was probed at 4 °C overnight in a humidified chamber. Each step was accompanied by three Tris-buffered saline (TBS) rinses, and the bound antibody on the array was detected by using SignalStain® Boost Detection Reagent (Cell Signaling Technology) and SignalStain® DAB (Cell Signaling Technology). Finally, sections were counterstained with Hematoxylin QS (VectorLaboratories) and the slide was mounted with VectaMount AQ (Vector Laboratories) for long-term preservation. CBFβ and CDK11 expressions were evaluated according to visible nuclear staining and calculated by the percentage of positive cells among the entire spot. Thereby, CBFβ staining patterns were categorized into 6 groups: 0, no nuclear staining; A, 1+, < 10% of cells stained positive; B, 2+, 10 to 25% positive cells; C, 3+, 26 to 50% positive cells; D, 4+, 51 to 75% positive cells; and E, 5+, > 75% positive cells. CBFβ and CDK11 staining images were obtained using a Nikon Eclipse microscope (Nikon Corp) with a SPOT RT digital camera (Diagnostic Instruments Inc.).

## Results

### Expression profiling of gene regulation by CDK11

**Fig. 1:**
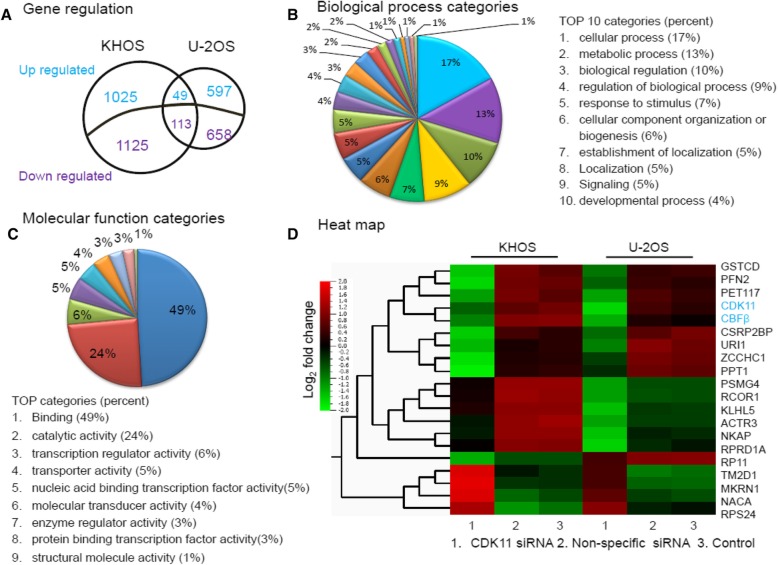
Global gene expression profiling reveals gene regulation in response to CDK11 knockdown. **a** Venn diagram of genes overexpressed and underexpressed in CDK11 siRNA treated cells compared with control group. Genes overexpressed/underexpressed in two cell lines are indicated in the overlapping regions of the circles. **b** Differentially expressed genes from gene expression profiling were grouped by biological process; the percentages of genes that are involved in each particular biological process are shown. **c** Distribution of molecular function was determined by differentially expressed genes (over 1.5-fold). **d** Heat map of significantly regulated genes in U-2OS and KHOS cell lines transfected with CDK11 siRNA. The genes listed in the hierarchical clustering represent the average log_2_ fold change

To identify the genes regulated in response to knockdown of CDK11 expression, KHOS and U-2OS osteosarcoma cell lines were transfected with CDK11 siRNA and their gene expression profiles were analyzed by Genesifter. The microarray data from this study has been submitted to the database of Gene Expression Omnibus (http://www.ncbi.nlm.nih.gov/geo/) and assigned the GEO accession number GSE73422 [26, 27]. Genome-wide gene profiling identified 162 differentially expressed genes (≥ 1.5 log fold alteration in expression, *P* < 0.05) in both KHOS and U-2OS cell lines with CDK11 siRNA knockdown (Fig. 1a and Additional file 2: Table S1), as compared with non-specific siRNA treated cells or untreated cells. Analysis by biological category revealed that these differentially expressed genes are enriched in cellular processes (17%), metabolic processes (13%), and biological regulation (10%) (Fig. 1b). Further analyses were focused on the molecular function of these differentially expressed genes. We observed that the genes involved in protein or DNA binding (*n* = 74), enzyme catalytic activity (*n* = 37), and transcription regulator activity (*n* = 9) comprised the most common molecular function categories (Fig. 1c). This indicated that CDK11 is involved in extensive biological regulation and plays important roles in transcription regulation. Further analysis of the altered gene clusters using Qlucore software revealed the top 20 significantly differentially expressed genes, which are shown in a heat map as Fig. 1d and Additional file 3: Table S2 (*P* < 0.05). Among these genes, 16 were downregulated, whereas the remaining genes were upregulated. CBFβ was the most highly correlated expressing gene with CDK11.

### CBFβ expression depends on CDK11 expression on transcriptional and translational levels

**Fig. 2:**
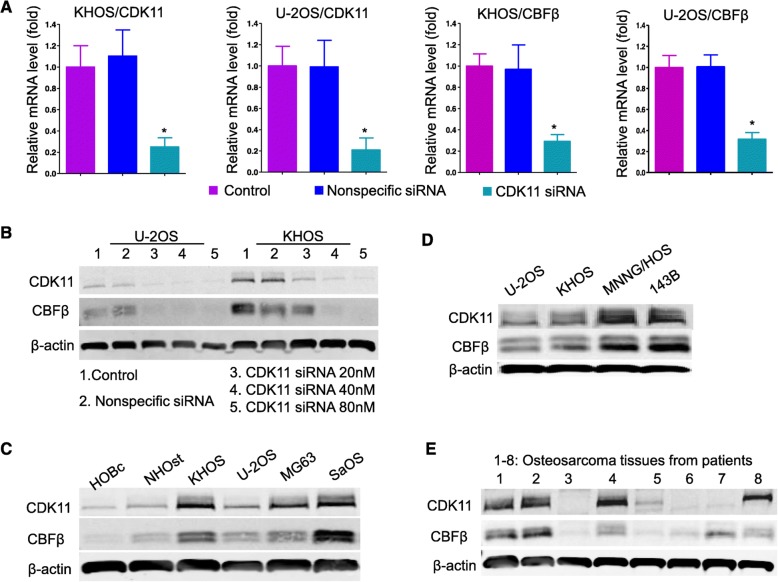
CBFβ expression depends on CDK11 expression on transcriptional and translational levels. **a** Verification of the altered gene expression observed in the gene array for selected genes, including CBFβ and CDK11, using quantitative RT-PCR. Two different cell lines (KHOS and U-2OS) were analyzed, expression was normalized using the KHOS or U-2OS untreated control, and results were depicted as relative expression in these cells. **b** CDK11 siRNA downregulated the expression of CDK11 and CBFβ in a dose-dependent manner. KHOS or U-2OS cells were transfected with CDK11 siRNA with increasing concentrations. The protein expression of CBFβ decreased dependently with CDK11 expression. **c** Expressions of CDK11 and CBFβ in osteosarcoma cell lines and in normal osteoblast cell lines. **d** Expressions of CDK11 and CBFβ in osteosarcoma cell lines and metastatic cell lines. **e** Expressions of CDK11 and CBFβ in osteosarcoma tissues. *P < 0.05

The relationship between CDK11 and CBFβ was evaluated in KHOS and U-2OS cell lines using quantitative RT-PCR. We observed knockdown of CDK11 significantly decreased transcript levels four-fold and five-fold in KHOS and U-2OS cell lines (Fig. 2a). In accordance with decreasing CDK11 mRNA, there was a three-fold decrease in CBFβ mRNA expression compared with controls in both KHOS and U-2OS cell lines (Fig. 2a). To investigate whether the downregulation in expression observed at the mRNA level was also reflected at the protein level, cells were transfected with increasing doses of CDK11 siRNA. CDK11 protein expression was suppressed by CDK11 siRNA in a dose-dependent manner, while CBFβ protein was also reduced in a dose-dependent manner (Fig. 2b). These results demonstrated that CBFβ expression depends on CDK11 expression at the translational level. In normal human cells, the expression of CDK11 is tightly regulated; for example, in the normal human osteoblast cell lines HOBc and NHOst, there are extremely low or almost undetectable levels of CDK11, while CBFβ levels are correspondingly low in normal human osteoblast cell lines and exhibit high expression in osteosarcoma cell lines (Fig. 2c). MNNH/HOS and 143B are osteosarcoma cell lines that are highly tumorigenic and are able to induce pulmonary metastases in xenograft mouse models in vivo [28, 29]. Interestingly, CDK11 expressions in MNNH/HOS and 143B cell lines were higher than in U-2OS and KHOS cell lines. Consistent with this, the protein expressions of CBFβ were also higher in the MNNH/HOS and 143B cell lines than the non-metastatic osteosarcoma U-2OS and KHOS cell lines (Fig. 2d). Four out of eight osteosarcoma tissue samples showed that both CBFβ and CDK11 proteins were highly expressed (Fig. 2E).

### CDK11^p110^ localizes to the nucleus and is required for the transcriptional regulation of CBFβ genes

**Fig. 3:**
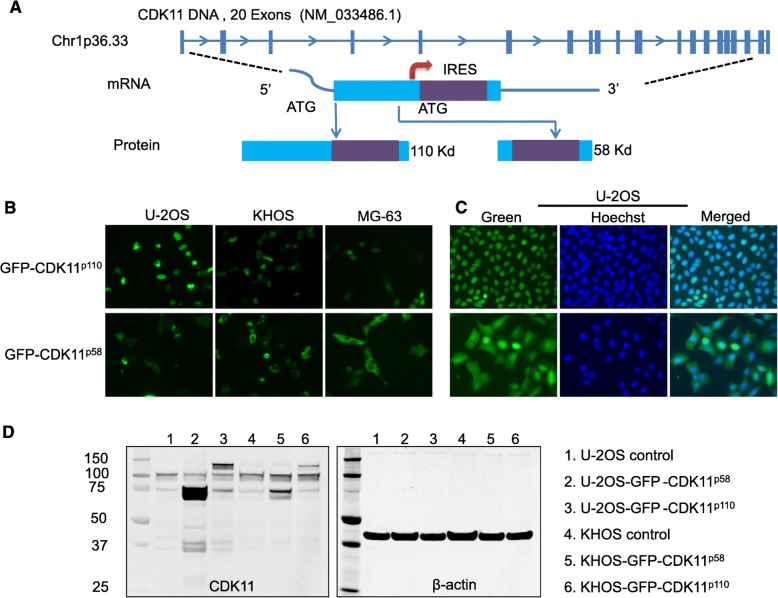
CDK11^p110^ localizes to the nucleus and exhibits high expression in tumor cells. **a** Schematic showing CDK11 chromosome exon locations, specifically the translated site that codes two protein isoforms. The CDK11^p58^ isoform is generated by an internal ribosome entry site sequence (IRES) in the same mRNA encoding the CDK11^p110^ isoform. **b** GFP-CDK11^p110^ and GFP-CDK11^p58^ transient transfection revealed that CDK11^p110^ is localized only in the nucleus, while CDK11^p58^ is expressed in the cytoplasm in three different cell lines (original magnification, 200×). **c** U-2OS-GFP-CDK11^p110^ and U-2OS- GFP-CDK11^p58^ selective stable cell lines show that CDK11^p110^ is localized only in the nucleus, while CDK11^p58^ is mainly expressed in the cytoplasm (original magnification, 200×). **d** CDK11^p110^ is highly expressed in U-2OS and KHOS cell lines; on the contrary, CDK11^p58^ is almost undetectable in these cell lines

The human CDK11 gene is located on chromosome 1p36.33 and contains 20 exons and 19 introns (Fig. 3a). To determine whether CDK11^p58^, CDK11^p110^, or both isoforms are associated with transcription, we first examined the subcellular localization of these two isoforms. Transfection with different CDK11 isomers fused with GFP expression vectors revealed that CDK11^p110^ was localized in the nucleus of three different osteosarcoma cell lines, while CDK11^p58^ was mainly localized in the cytoplasm (Fig. 3b). We then generated a U-2OS cell line stably expressing the GFP-CDK11^p58^ or the GFP-CDK11^p110^ protein (Fig. 3c). Consistent with transient transfection, the selected U-2OS- GFP-CDK11^p110^ cell line showed nuclear localization (Fig. 3c). Also as expected, the selected U-2OS-GFP-CDK11^p58^ cell line localized largely in the cytoplasm. Moreover, CDK11^p110^ was highly and constantly expressed in U-2OS and KHOS cell lines, whereas CDK11^p58^ was almost undetectable in these cells (Fig. 3d). Previous studies have shown that CDK11^p110^ protein is mainly localized in the nucleus of osteosarcoma cells both in cell lines and in tumor tissues [13]. Similar cellular localization of CDK11^p110^ has been demonstrated in other types of cancer cells, including in breast cancer and ovarian cancer [8, 9, 14]. Because CDK11^p110^ has been found broadly distributed onto chromatin and is involved in some aspects of transcription [20, 22], we investigated whether CBFβ was regulated by CDK11^p110^ or by CDK11^p58^.

**Fig. 4:**
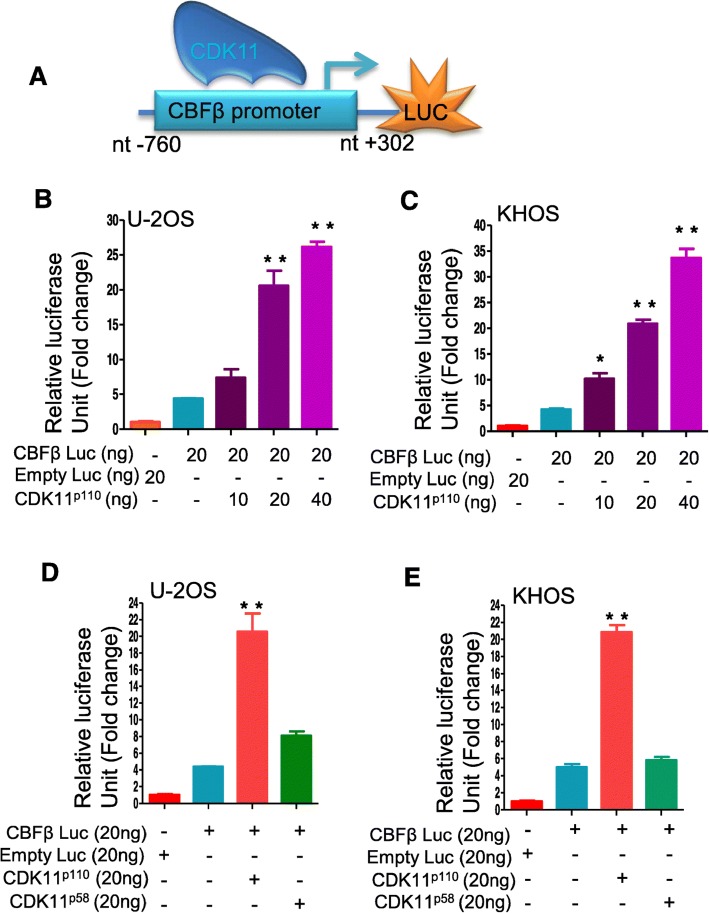
CDK11^p110^, not CDK11^p58^, regulates promoter activity of CBFβ genes. **a** Schematic representation of the promoter-luciferase construct showing the CBFβ promoter region. **b** CDK11 ^p110^ upregulates CBFβ promoter-luciferase in a dose-dependent manner in the U-2OS cell line. U-2OS cells were transfected with the CBFβ promoter-luciferase construct. Co-expression was conducted with an empty expression vector (control) or CDK11 expression vectors. Error bars indicate standard deviation and are from at least three replicates. **c** CDK11^p110^ upregulates CBFβ promoter-luciferase in a dose-dependent manner in the KHOS cell line. KHOS cells were transfected with the CBFβ promoter-luciferase construct. Co-expression was conducted with an empty expression vector (control) or CDK11 expression vectors. Error bars indicate standard deviation and are from at least three replicates. **d** CDK11^p110^, not CDK11^p58^, activates the CBFβ promoter-luciferase in the KHOS cell line. **e** CDK11^p110^ significantly increases CBFβ promoter-luciferase in KHOS cell line. **f** Schematic showing major structural features of the CDK11^p110^ protein. CDK11^p110^ kinase-dead or kinase-active mutations were generated. The asterisk represents the amino acid that was mutated to create kinase-dead or active mutations. **g** In the U-2OS cell line, the C-terminal kinase domain mutation of CDK11^p110^ failed to affect CDK11^p110^-mediated CBFβ activation. **h** The C-terminal kinase domain mutation of CDK11^p110^ also did not change CBFβ promoter activity in the KHOS cell line. *P < 0.05., **P < 0.01

We measured the regulatory activity of CDK11 on the CBFβ promoter using luciferase reporter assays. The length of the CBFΒ promoter region was 1062 nucleic acid base pairs (bp) from − 760 to + 302 relative to the transcriptional start site. To study how CDK11 regulates CBFβ expression, a DNA fragment containing this region was inserted upstream of the RenSP luciferase gene in the LightSwitch Promoter Reporter vector; once protein is bound to the CBFβ promoter region, it activates or enhances the luciferase activity (Fig. 4a). Transient transfection assays were conducted in KHOS and U-2OS cell lines. Co-expression of the CBFβ promoter luciferase vector with CDK11^p110^ increased luciferase activity seven-fold and six-fold in U-2OS and KHOS cells, respectively (Fig. 4b and c). Furthermore, CDK11^p110^ induced CBFβ promoter activity in a dose-dependent manner in both of the osteosarcoma cell lines. These results suggest that CDK11^p110^ acts as a transcriptional activator on the CBFβ promoter in these cell lines. We then investigated whether CDK11^p58^ was also involved in CBFβ regulation. As shown in Fig. 4d, in the presence of the CDK11^p110^ expression plasmid, CBFβ promoter activity was dramatically increased. However, the CDK11^p58^ expression vector had almost no effect on CBFβ promoter activity in the KHOS cell line. Similar to the KHOS cell line, CDK11^p58^ expression also showed no significant effect on CBFβ promoter activity in the U-2OS cell line (Fig. 4e). These data imply that CDK11^p110^, but not CDK11^p58^, increases CBFβ transcriptional activity.

### CDK11^p110^ directly upregulates CBFβ expression by binding to the CBFβ promoter

**Fig. 5:**
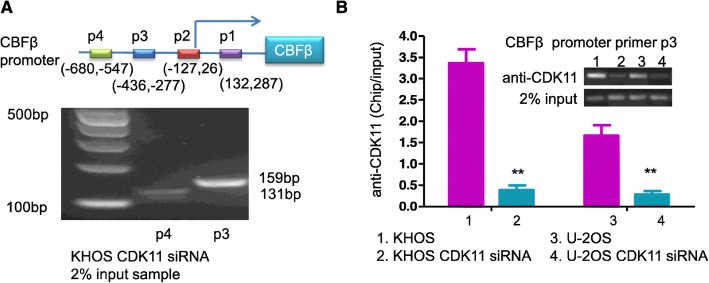
CDK11^p110^ upregulates CBFβ expression directly by associating with its promoter. **a** Schematic representation of potential CDK11 binding sites in the CBFβ promoter and primer sets (p1, p2, p3, p4) indicating amplified regions encompassing the four primer sites along with the transcription start site (TSS). Chromatin immunoprecipitations were analyzed using a 2% input of KHOS sample treated with CDK11 siRNA by PCR. PCR products were only observed with p3 and p4 primer. **b** ChIP analysis was performed by CDK11 antibodies or 2% input sample and by measuring enrichment at p3 in human CBFΒ promoter by RT-PCR. The amount of immunoprecipitated DNA by CDK11 antibodies are represented as ratio of input DNA (1:50) and presented as mean of three independent experiments (*n* = 3, mean ± SD). *P < 0.05; **P < 0.01, Student’s t-test. **c** Electrophoretic mobility shift assay of CDK11- CBFβ binding activity in nuclear extracts from different cell lines. Metastatic cell lines MNNH/HOS and 143B demonstrated notable high binding activity (lane 3 and 4, purple arrow) compared with KHOS and U-2OS non-metastatic cell lines. **d** The formation of CDK11-DNA complexes was determined by incubation with labeled CBFβ. Decreased CDK11 DNA-binding activity was present in CDK11 siRNA knockdown KHOS and MNNH/HOS cells (purple arrow)

DNA-protein interactions are crucial to fundamental cellular events, including gene transcription. Thus, these studies hold the key to our understanding of mechanisms underlying deregulated gene expression in human diseases, such as in cancer. The luciferase assay showed that CDK11^p110^ regulates the CBFβ promoter. However, whether this regulation was induced by CDK11^p110^ directly binding to the CBFβ promoter or not remains unclear. In order to identify the direct interaction between CDK11^p110^ and CBFβ, the chromatin immunoprecipitation (ChIP) assay was applied. The ChIP assay is a powerful method to study interactions between proteins and a specific genomic DNA region (protein-DNA interaction). Recruitment of proteins to promoters of specific genes is a common mechanism to activate or enhance gene transcription [30]. In the ChIP assay, the level of enrichment of the DNA sequence is determined relative to the total amount of input DNA (2% of input) (Additional file 1: Figure S1). To determine which site(s) might bind with the CDK11^p110^ protein, we designed four pairs of primers whose corresponding regions almost cover the whole length of the CBFβ promoter (− 680 to 287 bp) (Fig. 5a). Of the four pairs of primers, significant enhancement of CDK11^p110^ binding was observed by ChIP in Primer 3 (− 436/− 227) and Primer 4 (− 680/− 547) (Fig. 5a). Consistently, knockdown of CDK11^p110^ by siRNA significantly decreased the binding efficiency of the CBFΒ promoter region with Primer 3 (Fig. 5b). Sequencing of Primer 3 ChIP products confirmed that binding area located at the CBFβ promoter area in the genome (data were not shown). These data support an important role for CDK11^p110^ in mediating CBFβ expression in osteosarcoma through its ability to directly bind to the two unique sites of DNA sequences in the CBFβ promoter.

### CBFβ contributes to the proliferation and metastatic behavior of osteosarcoma cells

**Fig. 6:**
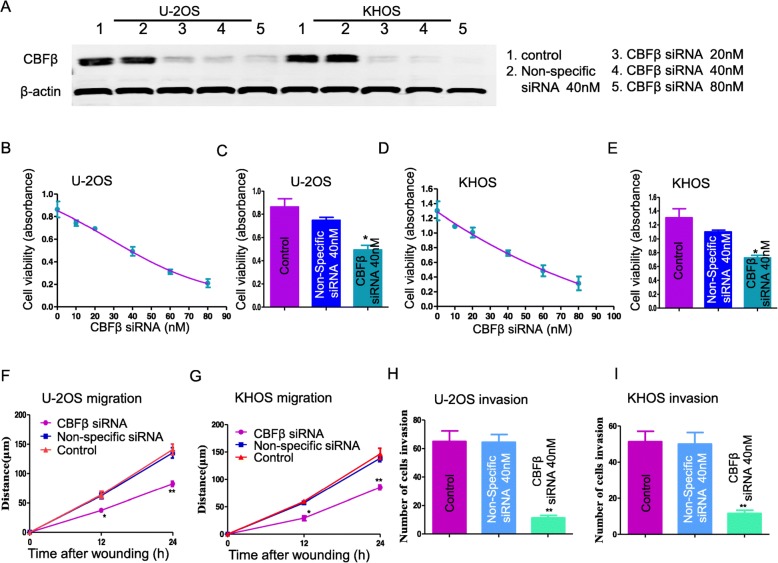
CBFβ contributes to the proliferation and metastatic behavior of osteosarcoma cells. **a** Confirmation of knockdown of CBFβ protein expression by Western blotting. CBFβ expressions were abrogated by CBFβ siRNA in a dose-dependent manner. **b** U-2OS cells were transfected with increasing concentrations of CBFβ siRNA, and cell proliferation after transfection was determined by MTT assay (*n* = 3). **c** U-2OS cells were transfected with CBFβ siRNA or non-specific siRNA, and cell proliferation after transfection was determined by MTT assay. **d** KHOS cells were transfected with increasing concentrations of CBFβ siRNA, and cell proliferation after transfection was determined by MTT assay. **e** KHOS cells were transfected with CBFβ siRNA or non-specific siRNA, and cell proliferation after transfection was determined by MTT assay. **f** Migration distance of U-2OS for each time point and condition. **g** Migration distance of KHOS for each time point and condition. **h** The average number of invasive U-2OS cells among those transfected with CBFβ siRNA or non-specific siRNA in 24 h. **i** The average number of invasive KHOS cells among those transfected with CBFβ siRNA or non-specific siRNA in 24 h. *P < 0.05; **P < 0.01 (comparison of transfected cells with control cells using Student’s t-test)

CDK11 plays a crucial role in cancer cell proliferation and growth, including in osteosarcoma. The current study has found that CDK11 protein can directly regulate CBFβ expression. Although CBFβ has been reported to play important roles during skeletal development in previous studies, the role of CBFβ in osteosarcoma are remains unknown. Therefore, we further investigated the functional role of CBFβ in osteosarcoma. Western blotting of cells with CBFΒ siRNA demonstrated that CBFβ expression can be inhibited in a dose-dependent manner (Fig. 6a). Depletion of CBFβ by siRNA resulted in dose-dependent cell growth inhibition in both KHOS and U-2OS cell lines, which was not observed with non-specific siRNA transfection (Fig. 6b to e). Since CBFβ showed higher expression in metastasis cell lines MNNH/HOS and 143B as compared with non-metastatic cell lines U-2OS and KHOS, we examined whether CBFβ knockdown by siRNA transfection could affect the migratory and invasive activities of osteosarcoma cells by wound healing assay and Matrigel invasion assay. The migration assay showed that downregulation of CBFβ significantly suppressed the migration of U-2OS and KHOS cells (Fig. 6f and g). Consistently, Matrigel invasion assays identified that the average numbers of osteosarcoma cells that were invading through the Matrigel after treatment with CBFβ siRNA were significantly lower than the blank control and the non-specific siRNA groups (Fig. 6h and i). Taken together, these results demonstrate that CBFβ plays a role in promoting osteosarcoma cell growth and invasion, similar to the roles of CDK11 in cancers [9, 11, 13, 15].

### High CBFβ expression correlates with CDK11 expression and contributes to reduced overall survival in osteosarcoma

**Fig. 7:**
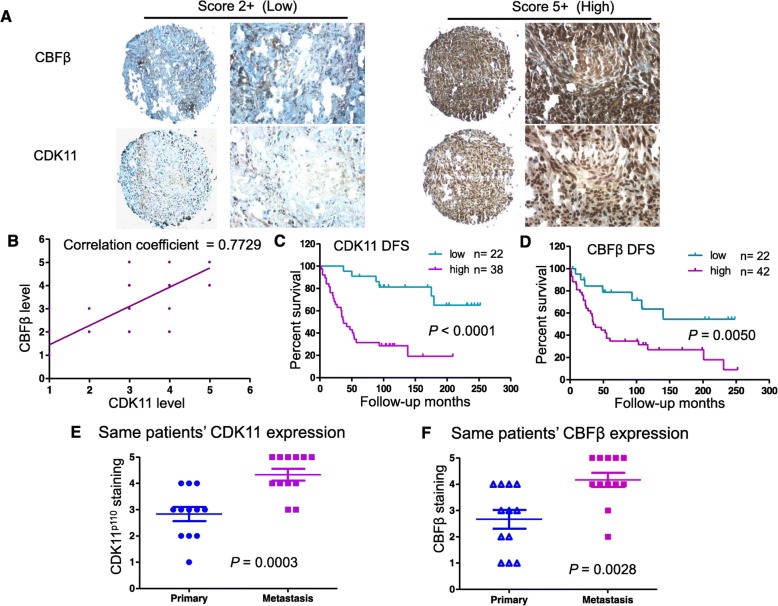
High CBFβ expression correlates with CDK11 expression and contributes to metastasis and reduced overall survival in patients. **a** Representative images of different immunohistochemical staining intensities of CBFβ and CDK11 staining are shown in osteosarcoma tissues. CBFΒ and CDK11 immunoreactivity was found mostly in the nucleus of tumor cells. The percentage of cells showing positive nuclear staining for CBFβ or CDK11 was calculated by reviewing the entire spot (original magnification, 200×). **b** The correlation coefficient (R^2^) between CBFβ expression and CDK11 expression was derived by GraphPad PRISM 5 (correlation coefficient R^2^ = 0.7729, *P* < 0.001). **c** Kaplan-Meier survival curve of patients with osteosarcoma were subgrouped as either CDK11 low staining (CDK11 staining ≤3) or high staining (CDK11 staining ≥4). **d** Kaplan-Meier survival curve of patients with osteosarcoma were subgrouped as either CBFβ low staining (CBFΒ staining ≤3) or high staining (CBFβ staining ≥4). **e** Distribution of CDK11 staining scores from the same patients with metastatic and primary specimens. **f** Distribution of CBFβ staining scores from the same patients with metastatic and primary specimens

CBFβ has been shown to contribute to malignancy in several human solid cancer types [31–34]. We further examined whether the levels of CDK11 correlated with the level of CBFβ in tumor cells by using a high-density tissue microarray (TMA), which included tissues from 73 available characteristics information of osteosarcoma specimens (Additional file 4: Table S3) [35]. Immunohistochemical analysis showed that CBFβ and CDK11 expression was quite variable in these samples. Among the evaluable specimens, 63.3% of CDK11 positive staining specimens were classified as CDK11-high patients (63.3%), and 65.6% of CBFβ positive staining specimens were categorized as CBFβ -high patients (Fig. 7a). These results indicated that the levels of CDK11 and CBFβ were highly correlated (correlation coefficient R^2^ = 0.7729, *P* < 0.0001, Fig. 7b). Kaplan-Meier analysis showed that osteosarcoma patients had a lower overall survival rate in the high CDK11 expression group compared with patients in the low CDK11 expression group (*P* < 0.0001) (Fig. 7c). Similarly, higher expression of CBFβ in osteosarcoma patients also predicted a significantly reduced survival rate (*P* = 0.0050) (Fig. 7d). Most importantly, the CDK11 expressions in 12 metastatic patients were dramatically higher in their metastatic samples than in their primary specimens (*P* = 0.0003) (Fig. 7e). Likewise, the CBFβ expressions in the samples of the metastatic patients were significantly higher than in their matched primary specimens (*P* = 0.0028) (Fig. 7f). The results above, coupled with the correlation of high CBFβ levels with metastatic behavior in breast, prostate, and ovarian cancer cells, support the notion that high CBFβ expression in the primary tumor may contribute to metastatic behavior.

## Discussion

In the current study, we have established that CBFβ is a CDK11 transcriptional target and promotes osteosarcoma cell growth and metastasis. We utilized global gene expression profiles after CDK11 knockdown by siRNA and discovered that CDK11 increases CBFβ -dependent transcriptional activation in osteosarcoma cells. Several protein DNA interaction experiments confirmed CBFβ as a direct transcriptional target of CDK11^p110^, but not CDK11^p58^. High expression of CBFβ was shown to be associated with poor outcome in osteosarcoma patients. This is the first report demonstrating a transcriptional regulation of CBFβ by CDK11 and that CBFβ can be a potential therapeutic target for osteosarcoma treatment.

Although CDK11 siRNA inhibits widespread transcription of a number of genes, CBFβ was the most significantly downregulated gene in the analysis. CBFβ, also known as Polyomavirus Enhancer-Binding Protein 2 Beta Subunit (PEBP2B), can positively regulate transcription from the RNA polymerase II promoter. CBFβ plays an indispensable role in skeletal development and homeostasis in various skeletal cell types [36–38]. Moreover, CBFβ is essential for invasion and xenograft tumor growth in some solid tumors, including in breast, prostate, and ovarian cancers [31, 32, 39, 40]. Therefore, further validations on the correlation between CDK11 and CBFβ expression were performed. There are at least three tiers of evidence that demonstrate that CDK11 mediates CBFβ pathway transcriptional regulation. Firstly, genome-wide gene profiling identified that CDK11 regulates CBFβ on a transcriptional level and that CBFβ protein expression depends on CDK11 protein expression. Secondly, promoter-luciferase assays demonstrated that CBFβ is regulated by CDK11^p110^, which is located only in the nucleus, while CDK11^p58^ is mainly expressed in the cytoplasm. Despite data clearly indicating that CDK11^p110^ interacts with transcriptional proteins and that it is required for high level in vitro transcription, the exact role of CDK11^p110^ in transcription in vivo and the biological substrates of the kinase remain undetermined [41]. Our results demonstrated that CDK11^p110^ induced CBFβ promoter activity in a dose-dependent manner in two osteosarcoma cell lines. Interestingly, we observed that CDK11^p110^, but not CDK11^p58^, increased CBFβ transcriptional activity. Consistent with this, CBFβ expression is not cell cycle-regulated and is maintained at high levels during the cell cycle in osteosarcoma cells [42]. Similarly, CDK11^p110^ expression is ubiquitous and constant throughout the cell cycle, while CDK11^p58^ is only specifically and transiently expressed during G2-M phase of the cell cycle. The structural features of CDK11^p110^ include its DNA binding area located on the N-terminus [21, 41, 43, 44]. BindN, a web-based tool for efficient prediction of DNA binding sites in amino acid sequences, predicted that 73.3% of DNA-binding residues are located on the N-terminus of CDK11^p110^ [45]. Thirdly, the ChIP assay confirmed that CBFβ is a direct transcriptional target of CDK11. Increasing evidence has revealed that the CDK family specifically occupies the promoters of a subset of genes [46–49]. In one study, ChIP confirmed CDK8 occupancy on the MYC promoter containing β-catenin/TCF elements [50]. Moreover, ChIP-seq demonstrated that CDK7 amplified MYCN mRNA and protein levels by occupying its promoter [51, 52]. Interestingly, CDK11 ^p110^ has been found broadly distributed on chromatin [22]. In addition, CDK11^p110^ has been proven to participate in transcription regulation initiated at an adenosine deaminase (GC)-rich promoter, while the CBFβ promoter region contains high GC content [20]. In this study, for the first time, we identified CBFβ as a direct CDK11 target gene and demonstrated that CDK11 binds to the proximal CBFβ promoter by ChIP assay. These data collectively verified that CBFβ is a sequence-specific target of CDK11^p110^ in human osteosarcoma cells. In the future, it will be of interest to determine whether this model applies to regulation by CDK11 in other systems.

In addition to demonstrating that CBFβ is a transcriptional target of CDK11, CBFβ showed similar biological functions to CDK11 in osteosarcoma. CDK11 is essential for cancer cell growth and invasion [9, 11, 13–16, 34]. In accordance with previous studies, we demonstrated that CBFβ is highly expressed in osteosarcoma cell lines, and especially highly expressed in osteosarcoma metastasis cell lines. We subsequently demonstrated that CBFβ depletion reduces cell viability, and migration and invasion activities of metastatic osteosarcoma cells. CDK11^p110^ expression is ubiquitous and constant throughout the cell cycle, and CBFβ is maintained at high levels during the cell cycle in osteosarcoma cells [42]. Aberrant expression of CBFβ proteins has been linked to pathological events in cancer cells [31, 32, 39]. Expression of CBFβ is essential for cancer cell invasion and migration, indicating that CBFβ may have an oncogenic function in tumor etiology [32, 39, 53–55]. Notably, expression levels of CDK11 and CBFβ protein are both extremely low in normal bone osteoblast cells, but are significantly higher in osteosarcoma cell lines. These may be explained by the fact that CDK11 and CBFβ possess similar essential roles in development. CDK11 knockout mice display an earlier lethality during the blastocyst stage of embryonic development [56]. Deletion of the CBFβ subunit revealed impaired bone formation and caused embryonic lethality [57].

CBFβ is the most frequently mutated and rearranged gene in human leukemias and plays an important role in hematologic malignancies [33, 55, 58]. Although no genetic defects have been reported for CBFβ in other types of human cancers, such as in osteosarcoma, there is a report of multiple CBFβ gene copies detected by FISH in a single case of granulocytic sarcoma associated with myeloid leukemia [59]. In line with previous gene profiling, high expression of CBFβ mRNA has been found in KHOS and U-2OS cell lines, and the expression of CBFβ is CDK11 expression dependent. Consistent with the correlation of CBFβ and CDK11 on the mRNA level, high CBFβ protein expression correlates with CDK11 expression and contributes to diminished overall survival. Furthermore, CDK11 and CBFβ expressions in metastatic patients were significantly higher than those in their matched primary specimens, which resulted in lower overall survival. Although CBFβ has been shown to play crucial roles in leukemia, the correlation between CBFβ and clinicopathological features has not been extensively analyzed in solid tumors. The precise role of CBFβ has not been fully elucidated, largely due to the complexity of the transcription factor itself. Since amplification and overexpression are generally considered hallmarks of an oncogene, CDK11 mediating CBFβ may be oncogenic.

## Conclusions

In conclusion, we have taken a genetic approach to understanding CDK11 regulated genes in osteosarcoma. A systematic combination of global gene expression profiling and DNA-protein assays has led to a wealth of information about how CDK11 and proteins interact in osteosarcoma cells. To our knowledge, no similar large-scale genetic analysis has been carried out on CDK11 or other members of CDK gene family. Our study identified CBFβ as a direct transcription target of CDK11^p110^. Importantly, we have found a new molecular mechanism that promotes osteosarcoma cell growth and provided preclinical evidence for the CDK11^p110^-mediated CBFβ pathway as a molecular marker to predict osteosarcoma metastasis risk. These results provide insights into the use of drugs that target CDK11^p110^- CBFβ for human osteosarcoma therapeutics.

## Supplementary information

**Additional file 1: Figure S1.** Crosslinked chromatin were digested and immunoprecipitated. (**A**) CDK11 siRNA-treated U-2OS and KHOS cells were formaldehyde-crosslinked and chromatin were prepared and digested by sonication into 1–5 nucleosomes in length (150–900 bp). (**B**) Chromatin immunoprecipitations were analyzed using the KHOS sample treated with CDK11 siRNA by PCR. PCR products were observed with RPL30 primer in Histone H3 Ab sample (lane 2) and input sample (lane 4), but not in the CDK11 antibody (Ab) sample (lane 1) and normal IgG ChIP sample (lane 3).

**Additional file 2: Table S1.** Top 162 gene list analyzed by Genesifter.

**Additional file 3: Table S2.** Top 20 gene list analyzed by Qlucore.

**Additional file 4: Table S3.** Characteristics of osteosarcoma patients and their tumor samples.

## Data Availability

The microarray data from this study has been submitted to Gene Expression Omnibus (http://www.ncbi.nlm.nih.gov/geo/) and assigned the GEO accession number GSE73422.

## Abbreviations

CBFβ: Core-binding factor subunit beta
CDK11: Cyclin-dependent protein kinase 11
ChIP: Chromatin immunoprecipitation
FBS: fetal bovine serum
GFP: Green fluorescent protein
IHC: Immunohistochemistry
MTT: 3-(4, 5-dimethylthiazolyl-2)-2, 5-diphenyltetrazolium bromide
siRNA: Small interfering RNA
TMA: Tissue microarray

## References

[1] Morgan DO (1995). Principles of CDK regulation. Nature.

[2] Murray AW (1994). Cyclin-dependent kinases: regulators of the cell cycle and more. Chem Biol.

[3] Palumbo A, Lau G, Saraceni M (2019). Abemaciclib: the newest CDK4/6 inhibitor for the treatment of breast Cancer. Ann Pharmacother.

[4] Formisano L, Lu Y, Servetto A, Hanker AB, Jansen VM, Bauer JA, Sudhan DR, Guerrero-Zotano AL, Croessmann S, Guo Y (2019). Aberrant FGFR signaling mediates resistance to CDK4/6 inhibitors in ER+ breast cancer. Nat Commun.

[5] Volkart PA, Bitencourt-Ferreira G, Souto AA, de Azevedo WF (2019). Cyclin-dependent kinase 2 in cellular senescence and Cancer. A structural and functional review. Curr Drug Targets.

[6] de Azevedo WF (2016). Opinion paper: targeting multiple cyclin-dependent kinases (CDKs): a new strategy for molecular docking studies. Curr Drug Targets.

[7] Evangelista M, Lim TY, Lee J, Parker L, Ashique A, Peterson AS, Ye W, Davis DP, de Sauvage FJ (2008). Kinome siRNA screen identifies regulators of ciliogenesis and hedgehog signal transduction. Sci Signal.

[8] Zhou Y, Han C, Li D, Yu Z, Li F, An Q, Bai H, Zhang X, Duan Z, Kan Q (2015). Cyclin-dependent kinase 11(p110) (CDK11(p110)) is crucial for human breast cancer cell proliferation and growth. Sci Rep.

[9] Kren BT, Unger GM, Abedin MJ, Vogel RI, Henzler CM, Ahmed K, Trembley JH (2015). Preclinical evaluation of cyclin dependent kinase 11 and casein kinase 2 survival kinases as RNA interference targets for triple negative breast cancer therapy. Breast Cancer Res.

[10] Jia B, Choy E, Cote G, Harmon D, Ye S, Kan Q, Mankin H, Hornicek F, Duan Z (2014). Cyclin-dependent kinase 11 (CDK11) is crucial in the growth of liposarcoma cells. Cancer Lett.

[11] Feng Y, Sassi S, Shen JK, Yang X, Gao Y, Osaka E, Zhang J, Yang S, Yang C, Mankin HJ (2015). Targeting CDK11 in osteosarcoma cells using the CRISPR-Cas9 system. J Orthop Res.

[12] Dos Santos Paparidis NF, Canduri F (2018). The emerging picture of CDK11: genetic, functional and medicinal aspects. Curr Med Chem.

[13] Duan Z, Zhang J, Choy E, Harmon D, Liu X, Nielsen P, Mankin H, Gray NS, Hornicek FJ (2012). Systematic kinome shRNA screening identifies CDK11 (PITSLRE) kinase expression is critical for osteosarcoma cell growth and proliferation. Clin Cancer Res.

[14] Liu X, Gao Y, Shen J, Yang W, Choy E, Mankin H, Hornicek FJ, Duan Z. Cyclin-dependent kinase 11 (CDK11) is required for ovarian Cancer cell growth in vitro and in vivo, and its inhibition causes apoptosis and sensitizes cells to paclitaxel. Mol Cancer Ther. 2016. 10.1158/1535-7163.MCT-16-0032 PMC4936930 27207777

[15] Zhou Y, Shen JK, Hornicek FJ, Kan Q, Duan Z. The emerging roles and therapeutic potential of cyclin-dependent kinase 11 (CDK11) in human cancer. Oncotarget. 2016. 10.18632/oncotarget.8519 PMC5130049 27049727

[16] Tiedemann RE, Zhu YX, Schmidt J, Shi CX, Sereduk C, Yin H, Mousses S, Stewart AK (2012). Identification of molecular vulnerabilities in human multiple myeloma cells by RNA interference lethality screening of the druggable genome. Cancer Res.

[17] Naik S, Dothager RS, Marasa J, Lewis CL, Piwnica-Worms D (2009). Vascular endothelial growth factor Receptor-1 is synthetic lethal to aberrant {beta}-catenin activation in Colon Cancer. Clin Cancer Res.

[18] Petretti C, Savoian M, Montembault E, Glover DM, Prigent C, Giet R (2006). The PITSLRE/CDK11p58 protein kinase promotes centrosome maturation and bipolar spindle formation. EMBO Rep.

[19] Housden BE, Valvezan AJ, Kelley C, Sopko R, Hu Y, Roesel C, Lin S, Buckner M, Tao R, Yilmazel B (2015). Identification of potential drug targets for tuberous sclerosis complex by synthetic screens combining CRISPR-based knockouts with RNAi. Sci Signal.

[20] Trembley JH, Hu D, Hsu LC, Yeung CY, Slaughter C, Lahti JM, Kidd VJ (2002). PITSLRE p110 protein kinases associate with transcription complexes and affect their activity. J Biol Chem.

[21] Hu D, Mayeda A, Trembley JH, Lahti JM, Kidd VJ (2003). CDK11 complexes promote pre-mRNA splicing. J Biol Chem.

[22] Drogat J, Migeot V, Mommaerts E, Mullier C, Dieu M, van Bakel H, Hermand D (2012). Cdk11-cyclinL controls the assembly of the RNA polymerase II mediator complex. Cell Rep.

[23] Porter S, Olson NE, Smith T. Analyzing gene expression data from microarray and next-generation dna sequencing transcriptome profiling assays using GeneSifter analysis edition. Curr Protoc Bioinformatics. 2009, Chapter 7:Unit 7 14 17 14 11–35. 10.1002/0471250953.bi0714s27. 10.1002/0471250953.bi0714s27 19728290

[24] Dunn W, Burgun A, Krebs MO, Rance B (2017). Exploring and visualizing multidimensional data in translational research platforms. Brief Bioinform.

[25] Bennett JP, Keeney PM (2018). RNA-sequencing reveals similarities and differences in gene expression in vulnerable brain tissues of Alzheimer's and Parkinson's diseases. J Alzheimers Dis Rep.

[26] Edgar R, Domrachev M, Lash AE (2002). Gene expression omnibus: NCBI gene expression and hybridization array data repository. Nucleic Acids Res.

[27] Djordjevic D, Tang JYS, Chen YX, Kwan SLS, Ling RWK, Qian G, Woo CYY, Ellis SJ, Ho JWK (2019). Discovery of perturbation gene targets via free text metadata mining in gene expression omnibus. Comput Biol Chem.

[28] Luu HH, Kang Q, Park JK, Si W, Luo Q, Jiang W, Yin H, Montag AG, Simon MA, Peabody TD (2005). An orthotopic model of human osteosarcoma growth and spontaneous pulmonary metastasis. Clin Exp Metastasis.

[29] Lamoureux F, Baud'huin M, Ory B, Guiho R, Zoubeidi A, Gleave M, Heymann D, Redini F (2014). Clusterin inhibition using OGX-011 synergistically enhances zoledronic acid activity in osteosarcoma. Oncotarget.

[30] Wagner M, Jung J, Koslowski M, Tureci O, Tiwari VK, Sahin U (2016). Chromatin immunoprecipitation assay to identify genomic binding sites of regulatory factors. Methods Mol Biol (Clifton, NJ).

[31] Davis JN, Rogers D, Adams L, Yong T, Jung JS, Cheng B, Fennell K, Borazanci E, Moustafa YW, Sun A (2010). Association of core-binding factor beta with the malignant phenotype of prostate and ovarian cancer cells. J Cell Physiol.

[32] Mendoza-Villanueva D, Deng W, Lopez-Camacho C, Shore P (2010). The Runx transcriptional co-activator, CBFbeta, is essential for invasion of breast cancer cells. Mol Cancer.

[33] Chin DW, Watanabe-Okochi N, Wang CQ, Tergaonkar V, Osato M (2015). Mouse models for core binding factor leukemia. Leukemia.

[34] Duployez N, Willekens C, Marceau-Renaut A, Boudry-Labis E, Preudhomme C (2015). Prognosis and monitoring of core-binding factor acute myeloid leukemia: current and emerging factors. Expert Rev Hematol.

[35] Gao Y, Feng Y, Shen JK, Lin M, Choy E, Cote GM, Harmon DC, Mankin HJ, Hornicek FJ, Duan Z (2015). CD44 is a direct target of miR-199a-3p and contributes to aggressive progression in osteosarcoma. Sci Rep.

[36] Chen W, Ma J, Zhu G, Jules J, Wu M, McConnell M, Tian F, Paulson C, Zhou X, Wang L (2014). Cbfbeta deletion in mice recapitulates cleidocranial dysplasia and reveals multiple functions of Cbfbeta required for skeletal development. Proc Natl Acad Sci U S A.

[37] Lim KE, Park NR, Che X, Han MS, Jeong JH, Kim SY, Park CY, Akiyama H, Kim JE, Ryoo HM (2015). Core binding factor beta of osteoblasts maintains cortical bone mass via stabilization of Runx2 in mice. J Bone Mineral Res.

[38] Yoshida CA, Furuichi T, Fujita T, Fukuyama R, Kanatani N, Kobayashi S, Satake M, Takada K, Komori T (2002). Core-binding factor beta interacts with Runx2 and is required for skeletal development. Nat Genet.

[39] Mendoza-Villanueva D, Zeef L, Shore P (2011). Metastatic breast cancer cells inhibit osteoblast differentiation through the Runx2/CBFbeta-dependent expression of the Wnt antagonist, sclerostin. Breast Cancer Res.

[40] Carlton AL, Illendula A, Gao Y, Llaneza DC, Boulton A, Shah A, Rajewski RA, Landen CN, Wotton D, Bushweller JH (2018). Small molecule inhibition of the CBFbeta/RUNX interaction decreases ovarian cancer growth and migration through alterations in genes related to epithelial-to-mesenchymal transition. Gynecol Oncol.

[41] Hu D, Lahti J, Choi S (2012). CDK11. Encyclopedia of signaling molecules.

[42] San Martin IA, Varela N, Gaete M, Villegas K, Osorio M, Tapia JC, Antonelli M, Mancilla EE, Pereira BP, Nathan SS (2009). Impaired cell cycle regulation of the osteoblast-related heterodimeric transcription factor Runx2-Cbfbeta in osteosarcoma cells. J Cell Physiol.

[43] Gururajan R, Lahti JM, Grenet J, Easton J, Gruber I, Ambros PF, Kidd VJ (1998). Duplication of a genomic region containing the Cdc2L1-2 and MMP21-22 genes on human chromosome 1p36.3 and their linkage to D1Z2. Genome Res.

[44] Feng Y, Qi W, Martinez J, Nelson MA (2005). The cyclin-dependent kinase 11 interacts with 14-3-3 proteins. Biochem Biophys Res Commun.

[45] Wang L, Brown SJ (2006). BindN: a web-based tool for efficient prediction of DNA and RNA binding sites in amino acid sequences. Nucleic Acids Res.

[46] Hou T, Ray S, Brasier AR (2007). The functional role of an interleukin 6-inducible CDK9.STAT3 complex in human gamma-fibrinogen gene expression. J Biol Chem.

[47] Orlando S, Gallastegui E, Besson A, Abril G, Aligue R, Pujol MJ, Bachs O (2015). p27Kip1 and p21Cip1 collaborate in the regulation of transcription by recruiting cyclin-Cdk complexes on the promoters of target genes. Nucleic Acids Res.

[48] Donner AJ, Szostek S, Hoover JM, Espinosa JM (2007). CDK8 is a stimulus-specific positive coregulator of p53 target genes. Mol Cell.

[49] Wagner AH, Conzelmann M, Fitzer F, Giese T, Gulow K, Falk CS, Kramer OH, Dietrich S, Hecker M, Luft T (2015). JAK1/STAT3 activation directly inhibits IL-12 production in dendritic cells by preventing CDK9/P-TEFb recruitment to the p35 promoter. Biochem Pharmacol.

[50] Firestein R, Bass AJ, Kim SY, Dunn IF, Silver SJ, Guney I, Freed E, Ligon AH, Vena N, Ogino S (2008). CDK8 is a colorectal cancer oncogene that regulates beta-catenin activity. Nature.

[51] Chipumuro E, Marco E, Christensen CL, Kwiatkowski N, Zhang T, Hatheway CM, Abraham BJ, Sharma B, Yeung C, Altabef A (2014). CDK7 inhibition suppresses super-enhancer-linked oncogenic transcription in MYCN-driven cancer. Cell.

[52] Kwiatkowski N, Zhang T, Rahl PB, Abraham BJ, Reddy J, Ficarro SB, Dastur A, Amzallag A, Ramaswamy S, Tesar B (2014). Targeting transcription regulation in cancer with a covalent CDK7 inhibitor. Nature.

[53] Zhao X, Chen A, Yan X, Zhang Y, He F, Hayashi Y, Dong Y, Rao Y, Li B, Conway RM (2014). Downregulation of RUNX1/CBFbeta by MLL fusion proteins enhances hematopoietic stem cell self-renewal. Blood.

[54] Greer AH, Yong T, Fennell K, Moustafa YW, Fowler M, Galiano F, Ng SW, Berkowitz RS, Cardelli J, Meyers S (2013). Knockdown of core binding factorbeta alters sphingolipid metabolism. J Cell Physiol.

[55] Hart SM, Foroni L (2002). Core binding factor genes and human leukemia. Haematologica.

[56] Li T, Inoue A, Lahti JM, Kidd VJ (2004). Failure to proliferate and mitotic arrest of CDK11(p110/p58)-null mutant mice at the blastocyst stage of embryonic cell development. Mol Cell Biol.

[57] Licht AH, Pein OT, Florin L, Hartenstein B, Reuter H, Arnold B, Lichter P, Angel P, Schorpp-Kistner M (2006). JunB is required for endothelial cell morphogenesis by regulating core-binding factor beta. J Cell Biol.

[58] Pulikkan JA, Hegde M, Ahmad HM, Belaghzal H, Illendula A, Yu J, O'Hagan K, Ou J, Muller-Tidow C, Wolfe SA (2018). CBFbeta-SMMHC inhibition triggers apoptosis by disrupting MYC chromatin dynamics in acute myeloid leukemia. Cell.

[59] Mallo M, Espinet B, Salido M, Ferrer A, Pedro C, Besses C, Perez-Vila E, Serrano S, Florensa L, Sole F (2007). Gain of multiple copies of the CBFB gene: a new genetic aberration in a case of granulocytic sarcoma. Cancer Genet Cytogenet.

