# Correction to: Transcriptional activation of CBFβ by CDK11^p110^ is necessary to promote osteosarcoma cell proliferation

**DOI:** 10.1186/s12964-019-0462-z

**Published:** 2019-10-29

**Authors:** Yong Feng, Yunfei Liao, Jianming Zhang, Jacson Shen, Zengwu Shao, Francis Hornicek, Zhenfeng Duan

**Affiliations:** 1 Department of Orthopaedic Surgery, Wuhan Union Hospital, Tongji Medical College, Huazhong University of Science and Technology, 1277 Jie Fang Avenue, Wuhan, 430022 China; 2 Sarcoma Biology Laboratory, Department of Orthopaedic Surgery, Department of Orthopaedic Surgery, David Geffen School of Medicine at UCLA, 615 Charles E. Young Dr. S, Los Angeles, CA 90095 USA

**Correction to: Cell Commun Signal (2019) 17:125**

**https://doi.org/10.1186/s12964-019-0440-5**

Following publication of the original article [1], it was reported that Figs. 4 and 5 were not updated during the production process.

The updated Figs. 4 and 5 are supplied below. The original article [1] has been corrected.

**Fig. 4:**
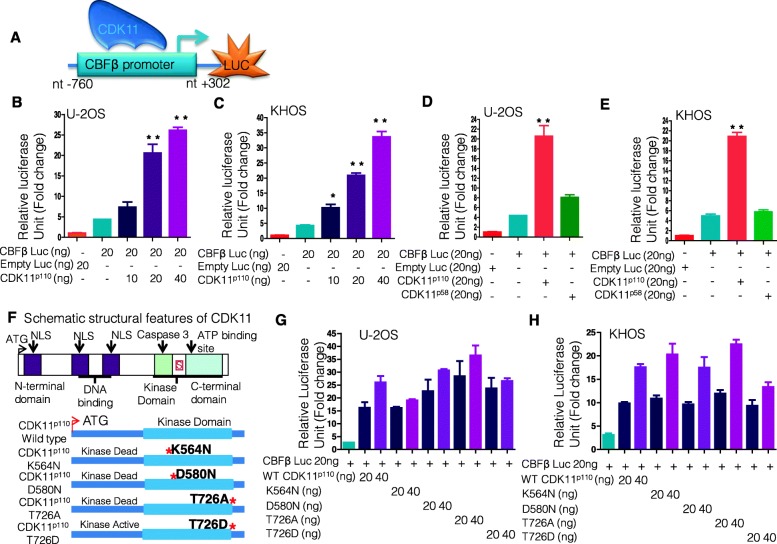
CDK11^p110^, not CDK11^p58^, regulates promoter activity of CBFβ genes. **a** Schematic representation of the promoter-luciferase construct showing the CBFβ promoter region. **b** CDK11 ^p110^ upregulates CBFβ promoter-luciferase in a dose-dependent manner in the U-2OS cell line. U-2OS cells were transfected with the CBFβ promoter-luciferase construct. Co-expression was conducted with an empty expression vector (control) or CDK11 expression vectors. Error bars indicate standard deviation and are from at least three replicates. **c** CDK11^p110^ upregulates CBFβ promoter-luciferase in a dose-dependent manner in the KHOS cell line. KHOS cells were transfected with the CBFβ promoter-luciferase construct. Co-expression was conducted with an empty expression vector (control) or CDK11 expression vectors. Error bars indicate standard deviation and are from at least three replicates. **d** CDK11^p110^, not CDK11^p58^, activates the CBFβ promoter-luciferase in the KHOS cell line. **e** CDK11^p110^ significantly increases CBFβ promoter-luciferase in KHOS cell line. **f** Schematic showing major structural features of the CDK11^p110^ protein. CDK11^p110^ kinase-dead or kinase-active mutations were generated. The asterisk represents the amino acid that was mutated to create kinase-dead or active mutations. **g** In the U-2OS cell line, the C-terminal kinase domain mutation of CDK11^p110^ failed to affect CDK11^p110^-mediated CBFβ activation. **h** The C-terminal kinase domain mutation of CDK11^p110^ also did not change CBFβ promoter activity in the KHOS cell line. *P < 0.05., **P < 0.01

**Fig. 5:**
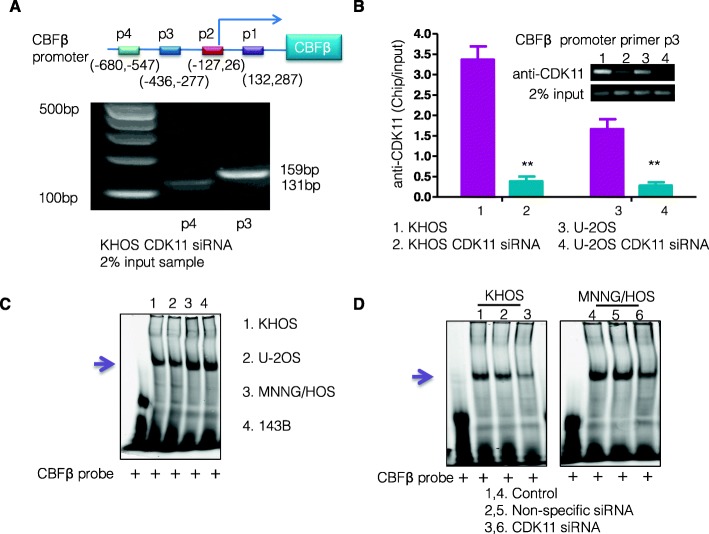
CDK11^p110^ upregulates CBFβ expression directly by associating with its promoter. **a** Schematic representation of potential CDK11 binding sites in the CBFβ promoter and primer sets (p1, p2, p3, p4) indicating amplified regions encompassing the four primer sites along with the transcription start site (TSS). Chromatin immunoprecipitations were analyzed using a 2% input of KHOS sample treated with CDK11 siRNA by PCR. PCR products were only observed with p3 and p4 primer. **b** ChIP analysis was performed by CDK11 antibodies or 2% input sample and by measuring enrichment at p3 in human CBFΒ promoter by RT-PCR. The amount of immunoprecipitated DNA by CDK11 antibodies are represented as ratio of input DNA (1:50) and presented as mean of three independent experiments (*n* = 3, mean ± SD). *P < 0.05; **P < 0.01, Student’s t-test. **c** Electrophoretic mobility shift assay of CDK11- CBFβ binding activity in nuclear extracts from different cell lines. Metastatic cell lines MNNH/HOS and 143B demonstrated notable high binding activity (lane 3 and 4, purple arrow) compared with KHOS and U-2OS non-metastatic cell lines. **d** The formation of CDK11-DNA complexes was determined by incubation with labeled CBFβ. Decreased CDK11 DNA-binding activity was present in CDK11 siRNA knockdown KHOS and MNNH/HOS cells (purple arrow)
