# Supplementary material for: Transcriptional activation of CBFβ by CDK11p110 is necessary to promote osteosarcoma cell proliferation
Source: Cell Commun Signal. 2019 Oct 14;17:125. doi: 10.1186/s12964-019-0440-5 (PMC6792216; doi:10.1186/s12964-019-0440-5)
Supplement: Supplementary file 1 — Additional file 1: Figure S1. Crosslinked chromatin were digested and immunoprecipitated. (A) CDK11 siRNA-treated U-2OS and KHOS cells were formaldehyde-crosslinked and chromatin were prepared and digested by sonication into 1–5 nucleosomes in length (150–900 bp). (B) Chromatin immunoprecipitations were analyzed using the KHOS sample treated with CDK11 siRNA by PCR. PCR products were observed with RPL30 primer in Histone H3 Ab sample (lane 2) and input sample (lane 4), but not in the CDK11 antibody (Ab) sample (lane 1) and normal IgG ChIP sample (lane 3). [file 12964_2019_440_MOESM1_ESM.pptx]

## Slide 1
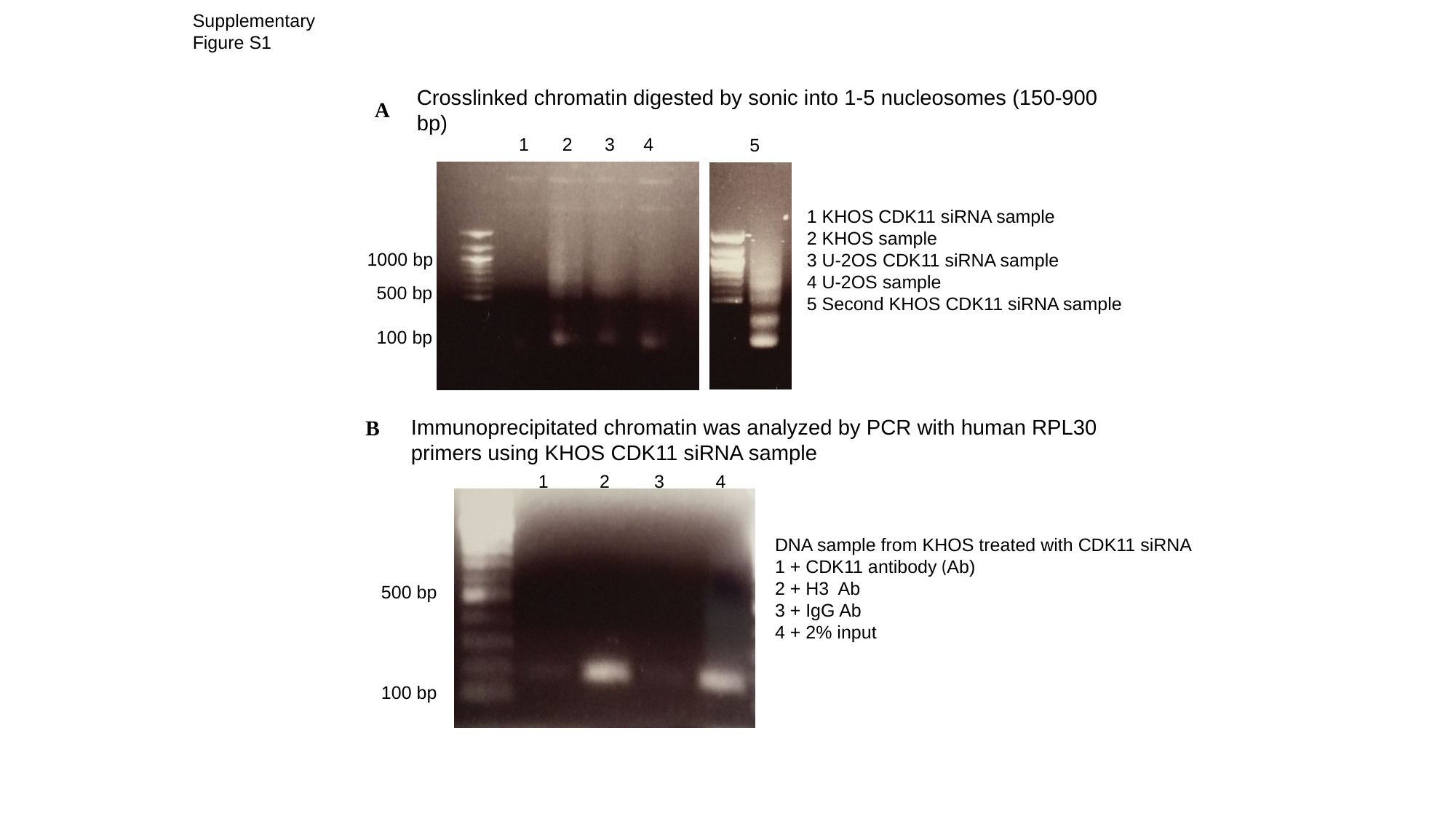

Supplementary Figure S1
Crosslinked chromatin digested by sonic into 1-5 nucleosomes (150-900 bp)
A
1
2
3
4
5
1 KHOS CDK11 siRNA sample
2 KHOS sample
3 U-2OS CDK11 siRNA sample
4 U-2OS sample
5 Second KHOS CDK11 siRNA sample
1000 bp
500 bp
100 bp
Immunoprecipitated chromatin was analyzed by PCR with human RPL30 primers using KHOS CDK11 siRNA sample
B
1
2
3
4
DNA sample from KHOS treated with CDK11 siRNA
1 + CDK11 antibody (Ab)
2 + H3 Ab
3 + IgG Ab
4 + 2% input
500 bp
100 bp
